# Robotic-assisted technology does not influence functional outcomes among obese and morbidly obese total knee arthroplasty patients

**DOI:** 10.1186/s40634-023-00634-8

**Published:** 2023-07-31

**Authors:** Brian P. McCormick, Sarah Trent, Xue Geng, Ji Won Lee, Henry R. Boucher

**Affiliations:** 1 Department of Orthopaedic Surgery, MedStar Union Memorial Hospital, 3333 North Calvert Street, Suite 400, Baltimore, MD 21218 USA; 2 Department of Orthopaedic Surgery, Donald and Barbara Zucker School of Medicine at Hofstra / Northwell, Great Neck, NY USA; 3 Department of Biostatistics, Georgetown University School of Medicine, Washington, D.C USA

**Keywords:** Total knee arthroplasty, Robotic-assisted, Obesity, Patient-reported Outcomes

## Abstract

**Purpose:**

Despite benefits of total knee arthroplasty (TKA) on function and quality of life, obese patients have less improved functional outcomes following TKA compared to their normal weight counterparts. Furthermore, obesity is a risk factor for aseptic loosening and revision surgery following TKA. With known benefits of robotic-assisted TKA (RaTKA) in precision and patient satisfaction, we aimed to evaluate the differences in patient reported outcome and early complication rates for patients undergoing RaTKA versus conventional TKA among patients of varying BMI groups.

**Methods:**

This study was a retrospective cohort study of patients who underwent conventional versus RaTKA. Patients were grouped by BMI range (< 30 kg/m2, 30–40 kg/m2, and > 40 kg/m2). Patient-reported outcomes were measured by Oxford Knee Scores and 12-Item Short Form Survey scores preoperatively, 6-month, 1-year, and 2-year postoperatively. Mixed-effects linear models were built for each patient-reported outcome to assess the interaction between type of surgery and BMI while adjusting for known confounders such as demographic variables.

**Results:**

A total of 350 patients (*n* = 186 RaTKA, *n* = 164 conventional TKA) met inclusion criteria. SF-12 physical scores were significantly higher at 2-year follow-up among non-obese patients compared to obese and morbidly obese patients (*p* = 0.047). There was no statistically significant interaction between the type of surgery performed (RaTKA versus conventional TKA) and obesity regarding their effects on patient reported outcomes.

**Conclusions:**

This study demonstrates no differences in functional outcomes among patients undergoing RaTKA compared to conventional TKA. Furthermore, obesity had no significant effect on this association.

**Level of evidence:**

III.

## Introduction

Obesity is a significant risk factor for knee osteoarthritis (OA) and  subsequent total knee arthroplasty (TKA) [1, 2]. Obese and morbidly obese patients have higher complication rates following TKA than their non-obese counterparts [3–5]. Postoperative wound complications are common among obese patients [3], and obesity has similarly been correlated with increased risk of aseptic loosening and revision surgery[6]. Component malpositioning such as tibial baseplate malpositioning is another risk factor for aseptic loosening, and the combination of component malpositioning and the presence of obesity has been shown to significantly increase the rate of revision surgery [7, 8]. With aseptic loosening being the most common indication for revision of a primary TKA in the obese population [6, 9], accurate implant positioning should be prioritized in order to mitigate this risk.

Robotic-assisted TKA (RaTKA) has been shown to improve component positioning with highly accurate bony resections compared to conventional TKA [10, 11]. Real-time feedback accounting for the patient’s unique bony anatomy and guidance from the robotic arm are designed to improve the precision and accuracy of bone cuts during TKA. Furthermore, benefits of RaTKA have translated to clinical outcomes as patients undergoing RaTKA have reported significantly higher patient satisfaction scores compared to patients undergoing conventional TKA [12].

The role of robotic-assisted surgery among obese patients undergoing TKA remains unclear. Previous studies have demonstrated significantly improved functional outcome scores for patients undergoing RaTKA compared to conventional TKA, although many prior studies did not specifically evaluate the relationship between obesity and robotic assistance [12, 13]. Studies have demonstrated improved function of obese patients after TKA compared to their preoperative status; however, these patients tend to have smaller improvements in function compared to non-obese patients undergoing TKA [3, 14, 15]. Additionally, a systematic review investigating the differences in perioperative and postoperative complications between RaTKA and conventional TKA with an administrative database found that obesity increased these risks [16]. Unfortunately, BMI was one of the high-risk factors that was controlled for in this study and therefore, it was not directly investigated. With evidence of benefits pertaining to RaTKA such as improved component positioning and patient satisfaction, it may be possible that these benefits may also be associated with obesity.

This study aimed to investigate the differences in postoperative outcomes for obese patients undergoing RaTKA versus conventional TKA. We hypothesized that robotic assistance during TKA will have a more significant effect on functional outcomes among obese and morbidly obese patients compared to their non-obese counterparts.

## Methods

The primary outcomes of this study were functional outcomes measured by Oxford Knee Scores (OKS) and 12-Item Short Form Survey (SF-12) scores. Secondary outcomes included postoperative complications and revision surgery rates evaluated through chart review of electronic medical records. Additionally, we sought to investigate whether obesity will moderate the effect of robotic assistance on outcomes following TKA.

### Study population and parameters

This study was a retrospective review of patients who underwent primary RaTKA or conventional TKA performed by a single fellowship-trained arthroplasty surgeon from January 1, 2015 to September 1, 2020. After Institutional Review Board approval (Study 00003377), potential subjects were identified using the institution’s OBERD, a patient reported outcomes (PRO) database. This database is utilized to collect patient-reported outcome measures (PROMs) as part of standard clinical follow-up. The OBERD system electronically sent surveys preoperatively and at 6-month, 1-year, and 2-year postoperative time points, and patients were contacted via telephone as necessary for incomplete PROMs. All patients 18 years of age or older who underwent RaTKA or conventional TKA with preoperative OKS and SF-12 scores documented in OBERD were eligible for the study. Patient consent was not obtained as this requirement was waived by IRB due to the retrospective nature of the study design.

Patients’ charts were reviewed using the institution’s electronic medical records to collect demographics (age, sex, body mass index (BMI), and procedure type), history of prior knee surgery, comorbidities recognized as part of the Elixhauser Comorbidity Index [17], and post-operative complications including deep vein thrombosis (DVT), pulmonary embolism (PE), arthrofibrosis requiring manipulation, periprosthetic fracture, and revision surgery.

### Robotic-assisted TKA surgical procedure

RaTKAs were performed using the Mako robotic system (Stryker, Kalamazoo, MI), a semi-active robotic arm with haptic saw guidance. A preoperative CT scan helped to generate a three-dimensional (3D) bone model and individualized surgical plan, which was then used intraoperatively. A standard medial parapatellar approach was used for exposure and tracker and reference pins were placed in the tibia and distal femur for registration. The robotic arm was used for sequential bone cuts, and implants were trialed and evaluated using the navigation system before final implant (Stryker Triathlon Cruciate-retaining knee, Mahwah, NJ) placement.

### Conventional TKA surgical procedure

Preoperative planning for conventional TKAs included standard multiplanar radiographs. The joint was exposed through a medial parapatellar approach with an intramedullary femoral guide for resecting the distal femur. The tibial cut was performed using an extramedullary guide. Flexion and extension gaps were then evaluated with spacer blocks and soft tissue releases were performed as necessary. Trial implants were inserted and evaluated for adequate balance before placement of final implants (Stryker Triathlon Cruciate-retaining knee, Kalamazoo, MI).

### Choice of RaTKA versus conventional TKA

Patients were presented with risks and benefits of each of the techniques. After this discussion between the patients and the surgeon, they collaboratively decided to proceed either with RaTKA or conventional TKA. Additionally, the selection of the procedure occurred regardless of any demographic variables such as age, weight, and/or comorbidities.

### Postoperative protocol

The postoperative protocol included pain management with multimodal medications intravenously and/or orally. Intravenous medication included the following alone or in combination: 1) morphine 1–2 mg, 2) dilaudid 1–2 mg, and/or 3) toradol 15 mg. Oral medication were one of the following alone or in combination: 1) oxycodone 5–10 mg, 2) hydrocodone/APAP 5–10 mg, 3) dilaudid 2–4 mg, 4) celebrex 200 mg, or 5) tramadol 50–100 mg.

Chemical DVT prophylaxis was used unless contraindicated. A hemovac drain was used intraoperatively and removed within 24 h after surgery. Postoperatively, patients were immediately weightbearing as tolerated with an assistive device such as a walker and began physical therapy the same day of the procedure for mobility and range of motion exercises. Physical therapy was strongly encouraged to be outpatient 2 to 3 times a week; however, it was arranged to be at home for those with transportation and/or mobility issues. All participants completed the stated protocol with a combination of both home and/or outpatient physical therapy. There was no difference in the rehabilitation protocol for both normal weight and obese patients. Patients followed up in the office at 4 weeks, 10 weeks, 1 year, and 2 years for radiographic and clinical evaluation. These timeframes differed from ones we used for collecting PROs, which were preoperatively, and at 6-month, 1-year, and 2-year postoperative time points.

### Patient reported outcomes

OKS and SF-12 scores were obtained by OBERD via emails, telephone, or during office visits preoperatively and postoperatively at 6 months, 1 year and 2 years from the time of index procedure. OKS is a validated set of 12 questions used to assess postoperative pain and function after TKA [18, 19]. Each question has a range of 0 to 4 for a summative score of 48, with 48 being the best possible score (least symptomatic), and 0 being the worst possible score (most symptomatic). SF-12 is also a validated set of 12 questions that measures quality of life (QoL) and is composed of two parts: a physical component summary and a mental component summary [19, 20]. Each component is reported on a 0 to 10 scale, with 0 indicating poorest QoL and 100 indicating best QoL.

### Statistical analysis

An a priori power analysis and sample size calculation was performed for the study to allow the study team to determine the number of patients that are required to detect the established MCID for OKS and SF-12 scores [21]. This provided a range of sample sizes based on varying degrees of MCID for the two scores. We determined that based on the sample size calculations, the midpoint of detecting 5 points for both OKS and SF-12 would be appropriate. This would also account for the correlation between time 1 and time 2 since the difference between time points were measured. Therefore, sample sizes of 54 for each cohort were determined necessary to achieve 81% power for detecting the MCID. A total sample size of 135 was therefore required after accounting for a 20% in loss to follow up.

Data were summarized by using frequencies and percentages for categorical variables, and means, standard deviations (SD), medians, and interquartile ranges (IQR) for continuous variables. Three longitudinally measured clinical outcomes (SF-12 Mental Score, SF-12 Physical Score, and OKS) were also displayed over four time points (pre-op, 6-months, 1-year, and 2-year) using box plots. Data normality was checked using the Shapiro–Wilk test. The percentage of data missing was generated for each variable. We imputed missing values depending on normality of the continuous variables. For normally distributed variables, we generated the mean and imputed the mean values to each of the missing values. Similarly for non-normally distributed variables, we generated the median and imputed the median values to each of the missing values.

Univariate association between type of surgery (robotic-assisted versus conventional) and patient-reported outcomes and between BMI categories (< 30 kg/m2, 30–40 kg/m2, and > 40 kg/m2) and patient-reported outcomes were evaluated at each time point (pre-op, 6-months, 1-year, and 2-year) using univariate linear regression. With these tests, we sought to investigate whether the type of surgery and/or BMI categories may separately have an effect on patient-reported outcomes. Based on data normality, two-sided t-test or Wilcoxon rank-sum test was used to compare continuous variables between two groups, and ANOVA or Kruskal–Wallis test was used for three group comparisons. Chi-square test or Fisher’s exact test was used to check the association between categorical variables.

Mixed-effects linear models were built for each patient-reported outcome (SF-12 Mental Score, SF-12 Physical Score, and OKS) to evaluate the effects of robotic-assisted surgery and BMI adjusting for demographic variables (age, sex, Elixhauser Comorbidity Index Score). Interaction between the type of surgery and BMI was added to the model to test whether BMI moderated the relationship between type of surgery and clinical outcomes. The interaction term then was removed from the model due to lack of statistical significance. The reference levels used for the mixed-effects linear model were the following: 1) time point as pre-operation, 2) sex as female, 3) type of surgery as Ra-TKA, and 4) BMI as < 30 kg/m^2^. Following the significant time point effect, follow-up pairwise comparisons among the mean SF-12 Mental Score preoperatively and at 2 years follow-up were conducted. Family-wise error rates across these tests (3 comparisons) were adjusted for comparisons to control for the false discovery rate approach as described by Benjamin and Hochberg [22]. A significance level of 0.05 was determined for statistical significance. All analyses were performed using the statistical software RStudio (Version 1.4.1106).

## Results

### Demographics

Summary of demographic variables for the entire sample is displayed in Table 1 and for the BMI subgroups (< 30 kg/m2, 30–40 kg/m2, and > 40 kg/m2) are displayed in Table 2. A total of 350 patients (*n* = 186 RaTKA and *n* = 164 conventional) met inclusion criteria. Patients in the RaTKA group were younger than those in the conventional group (65.1 ± 9.0 and 67.7 ± 8.7, respectively, *p* = 0.013). The Elixhauser Comorbidity Index (ECI) score was also lower in the RaTKA compared to the conventional TKA group (-0.9 ± 4.0 and 0.5 ± 4.8, respectively, *p* = 0.011). There was no statistically significant difference between the two groups for sex, BMI, or presence of prior surgery. As for the subgroups, the non-obese group was significantly older than both the obese and morbidly obese groups (68.92 ± 8.47 years, 64.97 ± 8.96 years, and 61.80 ± 7.64 years, respectively, *p* < 0.001). Similarly, the ECI score also demonstrated similar findings of higher ECI score in the non-obese group compared to the obese and morbidly obese groups (1.99 ± 4.06, -1.92 ± 3.71, and -2.05 ± 4.37, respectively, *p* < 0.001).

**Table 1:** Comparison of baseline demographics between conventional and Robotic-assisted (Ra) TKA cohorts

	**Conventional TKA** **(*n* = 164)**	**Ra-TKA** **(*n* = 186)**	***P* Value**
Age, years, mean (SD)	67.7 (8.7)	65.1 (9.0)	0.013
Female sex, n (%)	101 (61.6)	105 (56.5)	0.387
BMI, kg/m^2^, mean (SD)	31.8 (5.9)	31.9 (6.0)	0.941
ECI, mean (SD)	0.5 (4.8)	-0.9 (4.0)	0.011

^*^Values provided as mean and standard deviation

*BMI* Body mass index, *ECI* Elixhauser comorbidity index, *Ra-TKA* Robotic-assisted total knee arthroplasty

**Table 2:** Comparison of baseline demographics between BMI (BMI < 30, BMI 30–40, and BMI > 40) subgroups

	Non-obese (BMI < 30) (*n* = 154)	Obese (BMI 30–40) (*n* = 158)	Morbidly Obese (BMI > 40) (*n* = 38)	*P* Value
Age, year* (year)	68.92 (8.47)	64.79 (8.96)	61.80 (7.64)	< 0.001**
Female sex, n (%)	87 (56.5)	91 (57.6)	28 (73.7)	0.142
ECI*	1.99 (4.06)	-1.92 (3.71)	-2.05 (4.37)	< 0.001**

^*^Values provided as mean and standard deviation

^**^denotes statistical significance

*BMI* Body mass index, *ECI* Elixhauser comorbidity index, *Ra-TKA* Robotic-assisted total knee arthroplasty

Data normality was checked using the Shapiro–Wilk test. Preoperative OKS was found to be normally distributed, and all other analyzed variables were not normally distributed with a threshold of *p* < 0.05 for the Shapiro–Wilk test.

### Robotic-assisted surgery and obesity

There was no statistically significant interaction between the type of surgery (RaTKA versus conventional TKA) and the BMI groups (< 30 kg/m2, 30–40 kg/m2, and > 40 kg/m2), where there was pairwise comparison of BMI groups with < 30 kg/m2 as the reference group in the interaction. These comparisons (30–40 kg/m2 versus < 30 kg/m2 and > 40 kg/m2 versus < 30 kg/m2) were not statistically significant for SF-12 Mental Scores (B = -2.634, Standard Error (SE) = 1.381, *p* = 0.057 and B = 2.363, SE = 2.211, *p* = 0.285, respectively), SF-12 Physical Scores (B = 1.415, SE = 1.47, *p* = 0.985 and B = 0.933, SE = 2.300, *p* = 0.685, respectively), and OKS (B = 0.090, SE = 1.062, *p* = 0.932 and B = 1.021, SE = 1.699, *p* = 0.548, respectively). In short, obesity did not significantly moderate the effect of robotic assistance on PROMs.

### Oxford knee scores

Oxford knee scores were compared between the RaTKA and conventional TKA cohorts with the t-test preoperatively and with the Wilcoxon rank-sum test at the remaining time points (6 months post-op, 1 year post-op, and 2 years post-op). Similarly, these scores were compared among the different BMI groups (< 30 kg/m2, 30–40 kg/m2, and > 40 kg/m2) with ANOVA for preoperative scores and with Kruskal–Wallis test at the aforementioned remaining time points. There was no statistically significant difference in OKS between RaTKA and conventional TKA cohorts at all time points: preoperative (23.7 ± 8.4 and 23.6 ± 7.7, respectively, *p* = 0.877), 6 months (41.0, IQR: 40.0–41.8 and 40.0, IQR: 36.0–44.0, respectively, *p* = 0.896), 1 year (43.0, IQR: 42.0–45.8 and 41.0, IQR: 40.0–41.3, respectively, *p* = 0.355), and 2 years (45.0, IQR: 41.0–47.0 and 44.0, IQR: 39.0–47.0, respectively, *p* = 0.526) (Table 3). Non-obese patients had higher OKS preoperatively compared to obese and morbidly obese patients (25.3 ± 7.6, 22.9 ± 8.3, and 20.3 ± 7.3, respectively; *p* = 0.001) as demonstrated in Table 4. There was no significant difference in OKS among different BMI groups at the remaining postoperative time points: 6 months (41.0, IQR: 39.3–41.0; 41.0, IQR: 41.0–41.0; and 41.0, IQR: 40.0–44.0, respectively; *p* = 0.824), 1 year (43.0, IQR: 41.0–46.0; 43.0, IQR: 41.0–44.0; and 43.0, IQR: 39.3–47.0, respectively; *p* = 0.521), and 2 years (46.0, IQR: 41.0–47.0; 44.0, IQR: 40.0–47.0; and 44.0, IQR: 38.0–47.0, respectively; *p* = 0.214).

**Table 3:** Comparison of patient-reported outcome scores between conventional and Robotic-assisted (Ra) TKA

	**Conventional TKA** **(*n* = 164)**	**Ra-TKA** **(*n* = 186)**	***P* Value**
Oxford Knee Score			
Preoperative	23.7 (8.4)**	23.6 (7.7)**	0.887
6 month	40.0 (36.0–44.0)	41.0 (40.0–41.8)	0.896
1 year	41.0 (40.0–41.3)	43.0 (42.0–45.8)	0.355
2 year	44.0 (39.0–47.0)	45.0 (41.0–47.0)	0.526
SF-12 Physical			
Preoperative	29.6 (25.1–36.3)	31.5 (27.3–37.9)	0.096
6 month	44.6 (42.1–47.6)	44.6 (42.9–48.1)	0.780
1 year	49.4 (37.4–52.6)	49.4 (43.2–54.6)	*0.023
2 year	47.9 (36.6–54.8)	51.2 (39.6–55.5)	*0.020
SF-12 Mental			
Preoperative	55.4 (43.5–63.1)	56.6 (45.7–62.1)	0.903
6 month	57.8 (57.6–59.0)	57.8 (55.8–57.8)	0.079
1 year	56.3 (51.6–60.0)	56.3 (52.9–58.3)	0.370
2 year	57.8 (51.0–60.6)	57.3 (51.7–59.3)	0.415

Values provided in mean (SD) for ** and the rest are provided in median (IQR)

*SF-12* 12-item Short Form Survey, *Ra-TKA* Robotic-assisted total knee arthroplasty

^*^Denotes *p* < 0.05

**Table 4:** Comparison of patient-reported outcome scores between non-obese, obese, and morbidly obese cohorts

	**Non-obese (BMI < 30)** **(*n* = 154)**	**Obese (BMI 30–40)** **(*n* = 158)**	**Morbidly Obese (BMI > 40)** **(*n* = 38)**	***P* Value**
Oxford Knee Score				
Preoperative	25.3 (7.6)**	22.9 (8.3)**	20.3 (7.3)**	*0.001
6 month	41.0 (39.3–41.0)	41.0 (41.0–41.0)	41.0 (40.0–44.0)	0.824
1 year	43.0 (41.0–46.0)	43.0 (41.0–44.0)	43.0 (39.3–47.0)	0.521
2 year	46.0 (41.0–47.0)	44.0 (40.0–47.0)	44.0 (38.0–47.0)	0.214
SF-12 Physical				
Preoperative	32.0 (26.1–39.5)	30.0 (25.1–34.9)	28.7 (25.8–37.6)	*0.046
6 month	44.6 (42.8–48.1)	44.6 (42.1–45.7)	44.6 (40.3–52.5)	0.517
1 year	49.4 (42.1–55.1)	49.4 (41.9–52.7)	49.4 (37.2–55.2)	0.500
2 year	50.7 (40.0–55.9)	49.7 (37.0–54.6)	48.9 (38.1–54.5)	*0.047
SF-12 Mental				
Preoperative	57.0 (46.5–62.7)	55.4 (43.5–62.6)	51.5 (42.2–60.5)	0.244
6 month	57.8 (56.8–57.9)	57.8 (55.8–58.3)	57.8 (53.3–60.8)	0.907
1 year	56.3 (53.2–59.0)	56.3 (52.3–58.6)	56.3 (43.2–60.0)	0.346
2 year	57.8 (53.2–59.8)	57.4 (50.7–59.8)	57.0 (47.3–60.6)	0.425

Values provided in mean (SD) for ** and the rest are provided in median (IQR)

*SF-12* 12-item Short Form Survey, *BMI* Body mass index

^*^Denotes *p* < 0.05

### SF-12 physical scores

SF-12 Physical Scores were compared between the RaTKA and conventional TKA cohorts with the Wilcoxon rank-sum test at the different time points (preoperatively, 6 months post-op, 1 year post-op, and 2 years post-op). Similarly, these scores were compared among the different BMI groups (< 30 kg/m2, 30–40 kg/m2, and > 40 kg/m2) with Kruskal–Wallis test at the different time points.The mean SF-12 Physical Scores were significantly higher in the RaTKA versus conventional TKA cohort at 1 year follow-up (49.4, IQR: 43.2–54.6 and 49.4, IQR: 37.4–52.6, respectively; *p* = 0.023) and at 2-year follow-up (51.2, IQR: 39.6–55.5 and 47.9, IQR: 36.6–54.8, respectively; *p* = 0.020) as demonstrated in Table 3. There was no statistically significant difference in SF-12 Physical Scores between the two cohorts preoperatively (31.5, IQR: 27.3–37.9 and 29.6, IQR: 25.1–36.3, respectively; *p* = 0.096) or at 6 months follow up (44.6, IQR: 42.9–48.1 and 44.6, IQR: 42.1–47.6, respectively; *p* = 0.780). SF-12 physical scores were significantly higher in the non-obese cohort compared to obese and morbidly obese patients preoperatively (57.0, IQR: 46.5–62.7; 55.4 (43.5–62.6); and 51.5 (42.2–60.5), respectively, *p* = 0.046) and at 2-year follow-up (57.8, IQR: 53.2–59.8; 57.4, IQR: 50.7–59.8; and 57.0, IQR: 47.3–60.6, respectively, *p* = 0.047) as demonstrated in Table 4. There were no significant differences between these cohorts at 6 months (57.8, IQR: 56.8–57.9; 57.8, IQR: 55.8–58.3; and 57.8, IQR: 53.3–60.8, respectively, *p* = 0.517) and 1 year (56.3, IQR: 53.2–59.0; 56.3, IQR: 52.3–58.6; and 56.3, IQR: 43.2–60.0, respectively, *p* = 0.500).

### SF-12 mental scores

SF-12 Mental Scores were compared between the RaTKA and conventional TKA cohorts with the Wilcoxon rank-sum test at the different time points (preoperatively, 6 months post-op, 1-year post-op, and 2 years post-op). Similarly, these scores were compared among the different BMI groups (< 30 kg/m2, 30–40 kg/m2, and > 40 kg/m2) with Kruskal–Wallis test at the different time points. There was no statistically significant difference in SF-12 Mental Scores between RaTKA and conventional TKA cohort at all time points: preoperative (53.4 ± 10.9 and 53.3 ± 11.4, respectively, *p* = 0.903), 6 months (56.4 ± 6.5 and 55.9 ± 6.2, respectively, *p* = 0.079), 1 year (54.1 ± 9.0 and 54.8 ± 6.3, respectively, *p* = 0.370), and 2 years (54.6 ± 8.6 and 54.7 ± 7.9, respectively, *p* = 0.415) (Table 3). There was no significant difference in SF-12 Mental Scores between different BMI groups (< 30 kg/m2, 30–40 kg/m2, > 40 kg/m2) at all time points: preoperatively (57.0, IQR: 46.5–62.7; 55.4, IQR: 43.5–62.6; and 51.5, IQR: 42.2–60.5, respectively, *p* = 0.244), 6 months (57.8, IQR: 56.8–57.9; 57.8, IQR: 55.8–58.3; and 57.8, IQR: 53.3–60.8, respectively, *p* = 0.907), 1 year (56.3, IQR: 53.2–59.0; 56.3, IQR: 52.3–58.6; and 56.3, IQR: 43.2–60.0, respectively, *p* = 0.346), and 2 years (57.8, IQR: 53.2–59.8; 57.4, IQR: 50.7–59.8; and 57.0, IQR: 47.3–60.6, respectively, *p* = 0.425) (Table 4).

### Complications and revisions

The rates of complications and revisions were compared between the RaTKA and conventional TKA cohorts using Fisher’s exact tests. There was no significant difference in rates of postoperative complications (DVT: 0.6% and 1.1%, respectively, *p* = 0.637; and arthrofibrosis: 1.8% and 2.2%, respectively, *p* = 0.830) and revisions (1.8% and 0.6%, respectively, *p* = 0.533) between cohorts as demonstrated in Table 5. Additionally, one complication in the RaTKA cohort occurred among patients with BMI of < 30 kg/m2 and the rest were among those with BMI between 30–40 kg/m2. For conventional TKAs, two complications occurred among those with BMI between 30–40 kg/m2 group. All revisions in both the RaTKA and conventional TKA cohorts were among patients with BMI between 30–40 kg/m2.

**Table 5:** Comparison of complication rates between conventional and robotic-assisted TKA

	**Conventional TKA** **(*n* = 164)**	**Ra-TKA** **(*n* = 186)**	***P* Value**
Infection	0 (0.0)	0 (0.0)	-
DVT	2 (1.1)	1 (0.6)	0.637
Arthrofibrosis Requiring Manipulation	4 (2.2)	3 (1.8)	0.830
Revision Surgery	1 (0.6)	3 (1.8)	0.533

Values provided as n (%)

## Discussion

We found no statistically significant difference in OKS between the RaTKA and conventional TKA cohorts. Additionally, obesity did not moderate the association between robotic assistance and patient-reported outcomes. RaTKA was associated with improvement only in the SF-12 subscale pertaining to the physical component compared to conventional TKA, however, this difference may be considered negligible considering it may not yield to any appreciable clinical difference.

The use of robotic assistance during TKA is on the rise [23]. Studies have shown benefits of RaTKA compared to conventional TKA such as reduced postoperative pain, decreased hospital length of stay, and lower postoperative opioid requirements [24, 25]. Despite these advantages it is unclear whether robotic assistance is able to directly impact clinical and/or patient-reported outcomes of those with obesity. Previous studies have shown that young patients have higher rates of TKA failure and subsequent revision compared to older patients [26, 27]. It is possible that this potentially impacts patient-reported outcomes where younger patients may report different outcomes and/or function compared to their older counterparts. This may potentially bias results of this study where those in the RaTKA group were younger than those in the conventional group.

Several risk factors for aseptic loosening have previously been identified, including obesity, malalignment, and ligamentous imbalance [6, 7]. Importantly, the risk of aseptic loosening may increase with the combination of component malpositioning and obesity. Normal knee compartment pressure decreases from 10°, 45° to 90° [28] and a valgus femoral compartment may increase medial compartmental pressure while a varus one may increase lateral compartment pressure [29]. Additionally, internal and external rotation of the femoral component changes compartment pressures in flexion [29]. However, tibial component positioning is more sensitive than femoral component positioning with 1**°**-2**°** of valgus having an important influence over medial compartment pressure [30]. The same can be said to be true for varus placement and its influence in the lateral compartment [10]. The consequences of malalignment may be compounded by obesity and has been demonstrated in previous literature where there was a 168-fold increase in failure rates among patients with BMI greater than 33.7 kg/m2 [7]. Relatedly, robotic assistance in TKA has been shown to improve compartmental balancing in flexion [31]. This may possibly be due to measured resection of mechanical alignment, and an improvement in mechanical axis that may more reliably be placed in the center of the knee, which may dissipate forces equally. Furthermore, this may explain improved patient satisfaction in RaTKA and possibly result in longer survivorship than conventional TKA.

This study has several limitations. This is a single-surgeon study and results may not be generalizable to all arthroplasty surgeons. Furthermore, this study investigated the Stryker robotic assisted MAKO system and results may not be applicable to all types of RaTKA. Further research is warranted to understand the relative indications for robotic assistance during TKA such as the surgeon’s experience and volume by accounting for these variables or including participants who had TKA performed by different surgeons, which may increase the generalizability of our findings. Although our primary outcomes related to patient-reported outcomes, parameters such as length of stay, postoperative pain, or opioid consumption may have also been considered as additional outcome variables. Future studies investigating short-term outcomes of RaTKA and obesity should consider these variables as possible variables of interest. This is a retrospective study and inherently limited. Furthermore, the decision to proceed with either robotic-assisted or conventional TKA was a shared decision between the surgeon and the patient. This may have introduced selection bias due to inherent patient characteristics that may have altered the choice for robotic-assisted versus conventional TKA. Additionally, our choice for determining BMI ranges for subgroups was based on the Principal Investigator’s clinical observation, which may have inherently biased our findings. Future research regarding BMI and outcomes among patients who underwent total knee arthroplasty may focus on providing a clearer guideline for stratifying BMI risk groups. Another limitation of our study was the lack of sample size that limited our methods. Advanced statistical methods (i.e., propensity score matching) could have been considered for our study; however, we were unable to employ such methods due to lack of sample size with stratification based on BMI. Moreover, lack of evidence and/or guidelines regarding how to determine these BMI subgroups did not allow us an adequate way of performing these power analyses and sample size calculations. Since power analysis may also depend on the surgeon’s experience and/or learning curve, we may consider conducting a future study where we may estimate our sample size based on our Principal Investigator’s specific effect size derived from this study and his particular learning curve. There may have also potentially been bias due to differences in age and/or comorbidities between those who had robotic-assisted versus conventional TKA. Future studies may consider the influence of certain demographic variables such as age or comorbidities when conducting similar comparative studies.

## Conclusion

This study demonstrates no statistically significant differences in functional outcomes among patients undergoing RaTKA compared to conventional TKA. Moreover, obesity had no significant effect on this association. Surgeons may consider robotic assistance in patients undergoing TKA in light of our study findings that demonstrated non-inferior effects of RaTKA on PROMs compared to conventional TKA. Furthermore, though we did not find significant associations between obesity on PROMs, surgeons may continue to proceed with caution when performing TKAs, either robot-assisted or conventional, on obese patients.

## Data Availability

All data included in this study was collected securely and de-identified data is available upon reasonable request.
